# Supplementary material for: Predicting visual function by interpreting a neuronal wiring diagram
Source: Nature. 2024 Oct 2;634(8032):113–23. doi: 10.1038/s41586-024-07953-5 (PMC11446822; doi:10.1038/s41586-024-07953-5)
Supplement: Supplementary file 6 — CRF and ERF predictions for individual TmY4 and TmY9 cells. Analogous to Supplementary Data 3, but for TmY target types. Shown are the top four monosynaptic pathways, the strongest pathway passing through each of the top ten intermediary types (ranking from Extended Data Fig. 7), and the trisynaptic pathway Tm1–TmY–Dm3–TmY (see the section entitled Prediction of spatial normalization). [file 41586_2024_7953_MOESM6_ESM.zip › DataS4/TmY4/720575940623115802.pdf]

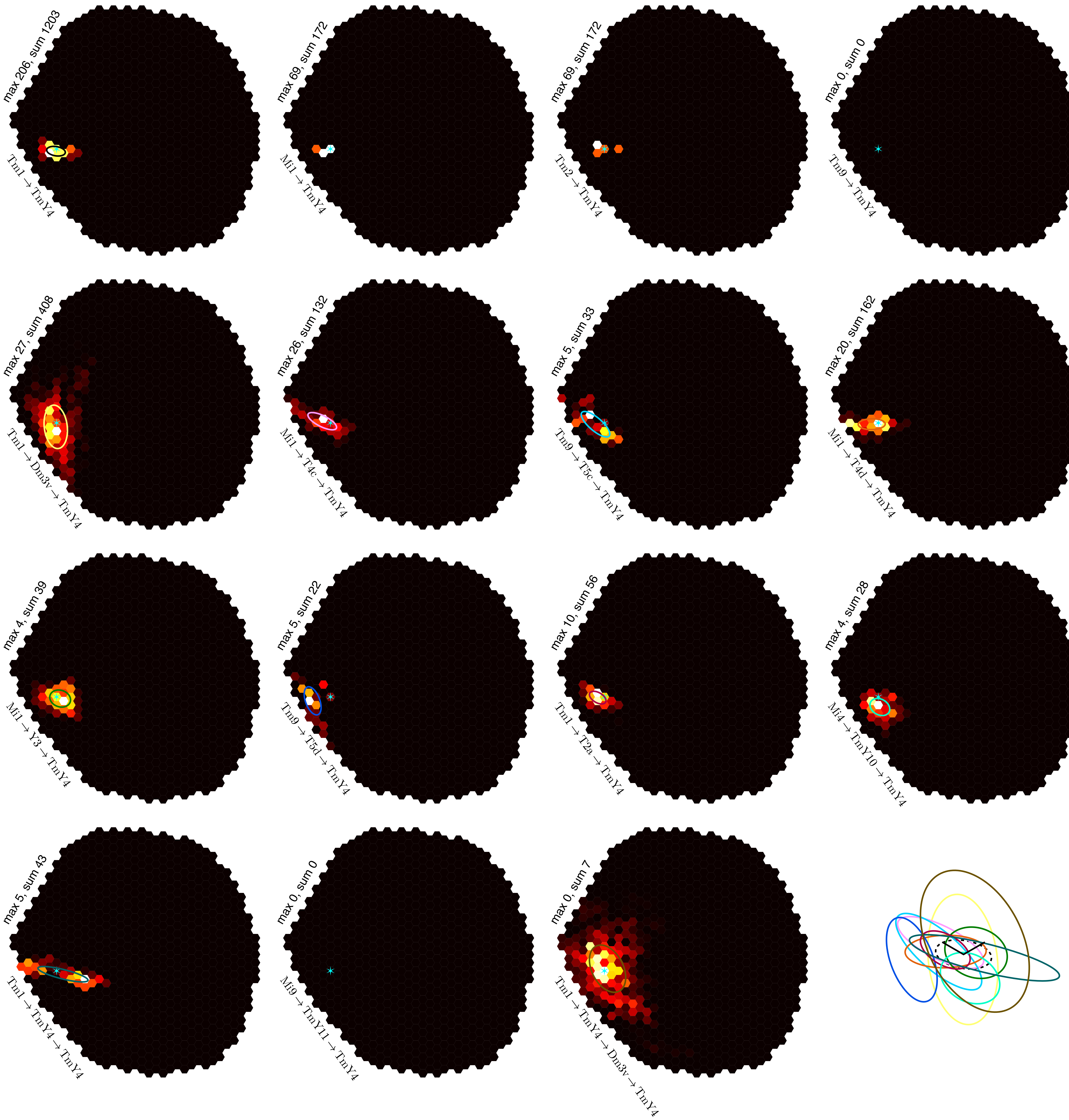
max 206, sum 1203
Tm1 → TmY4
max 69, sum 172
Mi1 → TmY4
max 69, sum 172
Tm2 → TmY4
max 0, sum 0
Tm9 → TmY4
max 27, sum 408
Tm1 → Dm3v → TmY4
max 26, sum 132
Mi1 → T4c → TmY4
max 5, sum 33
Tm9 → T5c → TmY4
max 20, sum 162
Mi1 → T4d → TmY4
max 4, sum 39
Mi1 → Y3 → TmY4
max 5, sum 22
Tm9 → T5d → TmY4
max 10, sum 56
Tm1 → T2a → TmY4
max 4, sum 28
Mi4 → TmY10 → TmY4
max 5, sum 43
Tm1 → TmY4 → TmY4
max 0, sum 0
Mi9 → TmY11 → TmY4
max 0, sum 7
Tm1 → TmY4 → Dm3v → TmY4
